# Squamous Cell Carcinoma Biomarker Sensing on a Strontium Oxide-Modified Interdigitated Electrode Surface for the Diagnosis of Cervical Cancer

**DOI:** 10.1155/2019/2807123

**Published:** 2019-04-07

**Authors:** Hongqing Wang, Thangavel Lakshmipriya, Yeng Chen, Subash C. B. Gopinath

**Affiliations:** ^1^Department of Gynecology, Shandong Provincial Hospital affiliated to Shandong University, No. 324 Jingwu Road, Huaiyin District, Jinan, Shandong Province 250021, China; ^2^Institute of Nano Electronic Engineering, Universiti Malaysia Perlis, 01000 Kangar, Perlis, Malaysia; ^3^Department of Oral & Craniofacial Sciences, Faculty of Dentistry, University of Malaya, 50603 Kuala Lumpur, Malaysia; ^4^School of Bioprocess Engineering, Universiti Malaysia Perlis, 02600 Arau, Perlis, Malaysia

## Abstract

Cervical cancer is a life-threatening complication, appearing as the uncontrolled growth of abnormal cells in the lining of the cervix. Every year, increasing numbers of cervical cancer cases are reported worldwide. Different identification strategies were proposed to detect cervical cancer at the earlier stages using various biomarkers. Squamous cell carcinoma antigen (SCC-Ag) is one of the potential biomarkers for this diagnosis. Nanomaterial-based detection systems were shown to be efficient with different clinical biomarkers. In this study, we have demonstrated strontium oxide-modified interdigitated electrode (IDE) fabrication by the sol-gel method and characterized by scanning electron microscopy and high-power microscopy. Analysis of the bare devices indicated the reproducibility with the fabrication, and further pH scouting on the device revealed that the reliability of the working pH ranges from 3 to 9. The sensing surface was tested to detect SCC-Ag against its specific antibody; the detection limit was found to be 10 pM, and the sensitivity was in the range between 1 and 10 pM as calculated by 3*σ*. The specificity experiment was carried out using major proteins from human serum, such as albumin and globulin. SCC-Ag was shown to be selectively detected on the strontium oxide-modified IDE surface.

## 1. Introduction

Cervical cancer is a gynecological issue originating from the cervix due to tissue invasiveness in the uterine cervix by uncontrolled cell division [1]. Cervical cancer is a burden to women's health and is the second leading cause of cancer-related death globally, accounting for ~250,000 cases every year [1–3]. Cervical cancer is generally caused by infection of the human papillomavirus (HPV), a common viral infection reported widely [4]. Almost all cervical cancer cases are attributable to the HPV infection, and untreated or undetected cases will result in life-threatening illness. Identifying cervical cancer at an earlier stage with suitable biomarkers will aid in the treatment of this complication and avoid disease spread to other organs [5].

Most cervical cancer cases are of squamous cell type origin [6]. Squamous cell carcinoma antigen (SCC-Ag) is a tumor antigen of the TA-4 subfraction and is a recognized prognostic factor for squamous cell cervical carcinoma (SCC) [7]. It was found that 95% of SCC occur in the uterine cervix, neck, and lung [8–10], and 83% are detected in the region of the uterine cervix. Thus, SCC-Ag is a well-proven biomarker for cervical cancer and has been identified to have a higher association between cervical cancer and SCC [11–13]. Looi et al. [14] have analyzed suitable biomarkers for cervical cancer by comparing the glycoprotein CA125 and SCC-Ag. The levels of CA125 and SCC-Ag were determined in the patients using the enzyme-linked immunosorbent assay; they found elevation of SCC-Ag in the serum. Their research concluded that SCC-Ag is a better tumor biomarker than CA125 to detect cervical cancer efficiently.

Because SCC-Ag is a suitable biomarker for cervical cancer, identifying and quantifying the critical level of SCC-Ag are mandatory to detect and treat cervical cancer. Creating a high-performance biosensor with a lower limit of SCC-Ag detection will aid the treatment of cervical cancer patients. The detection limit of any given biosensor is mainly dependent on two factors, the binding affinity between the probe and analyte and making proper surface functionalization without noticeable biofouling [15, 16]. In the past, probes including DNA, RNA, antibody, peptide, and aptamer have been considered to detect diseases with improved detection limits [17–20]. Surface functionalization for biomolecular attachment is highly associated with good biosensors, especially if it has been proven that high and proper arrangements of the analytes or molecules to be detected on the sensing surfaces enhance the limits of detection. Different chemical and physical methods were demonstrated either with a linker or directly immobilized to the biomolecules on the sensing surfaces. Among them, nanoparticles have attracted greater attention in the field of biosensors to improve detection due to nanoparticle-mediated enhancement of the surface area [15, 21–23].

Various nanomaterials have been used in the field of biosensors, especially in the medical field, for imaging, targeting, identification and targeted therapy. Among different nanomaterials, nanoparticles have attracted more attention for creating high-performance biosensors. Nanoparticles, including gold, silver, iron, copper, and graphene, have been used with appropriate surface functionalization to conjugate the desired probe [24, 25]. Additionally, strontium is a developing material that has been applied in different fields of interest, including as a biosensor [26–28]. Strontium is a widely used attractive perovskite type of material due to its different chemical and physical properties, as well as its applications in an electronic field as a capacitor and catalysis [29]. In this work, we used strontium oxide for surface functionalization to specifically detect SCC-Ag, and the desired strontium oxide surface is made on interdigitated electrode (IDE) that was immobilized with an SCC-Ag anti-antibody. The primary role of this research was to generate a sensor that can demonstrate high-performance detection with significant nonfouling and that can mimic for other clinically important targets. The significance of this research is to enhance the detection level of SCC-Ag to generate an early detection strategy that is expected to play a pivotal role in decreasing mortality due to SCC.

## 2. Materials and Methods

### 2.1. Reagents and Biomolecules

SCC-antigen was procured from RANDOX Life Sciences (Malaysia). Anti-SCC antibody was purchased from Next Gene (Malaysia). Strontium oxide, (3-aminopropyl)triethoxysilane (APTES), globulin, serum albumin, and Tween 20 were purchased from Sigma Aldrich (USA). All obtained reagents and chemicals were stored according to the manufacturers' recommendations. All pH solutions were from Hanna Instruments (Malaysia).

### 2.2. Fabrication of the IDE Sensor Surface

The IDE fabrication process was followed as described previously with different physical modifications on the top layer [18]. Briefly, after oxidation of the silicon wafers at high temperature, the etching process was performed using aluminum. Following etching, other developing processes were carried out similar to those in our earlier procedure. Finally, the strontium oxide layer was overlaid on the top for ultimate biomolecular immobilization. Before proceeding with strontium oxide attachment, both the sensing surface and strontium oxide were initially washed with 1 M potassium hydroxide (pH 9.0). To immobilize the strontium oxide on the IDE sensor surface, it was first mixed with 2% APTES (diluted in 70% ethanol) and kept at room temperature (RT) for 3 hrs. After the binding of strontium oxide and APTES, the unbound molecules were separated by centrifugation at 10,000 × g for 10 min. The APTES bound strontium oxide sediment was washed 3 times with 70% ethanol, followed by washing with water and immobilization of the antibody on the IDE sensor surface.

### 2.3. Antibody Immobilization on the IDE Sensor Surface via Strontium Oxide

The antibody was immobilized on the IDE sensor surface using the following steps. After performing the above steps with strontium oxide attachment, 2% glutaraldehyde (GLU) diluted in PBS was added onto the APTES-strontium oxide-modified surface and incubated for 1 h at RT. Next, the surface was washed 3 times with PBS to remove unbound GLU. On the GLU-modified surface, 200 nM of SCC-Ag antibody diluted in PBS was added, followed by incubation for 1 h at RT and washing 3 times with PBS to remove the unbound antibody. Finally, 1 M ethanolamine was added onto the remaining IDE surface to reduce biofouling.

### 2.4. Detection of SCC-Ag on the Strontium Oxide-Antibody-Modified IDE Surface

SCC-Ag was detected on the strontium oxide antibody-modified IDE sensor surface. First, the antibody was immobilized on the sensor surface using the above method. The baseline current was noted at this point. Thereafter, 5 *μ*l of 100 nM SCC-Ag was dropped onto the surface, followed by incubation for 30 min at RT and washing 3 times with PBS to remove unbound SCC-Ag. The changes in the current levels indicate the interaction of the antibody and SCC-Ag.

### 2.5. Limit of Detection: SCC-Ag on the Strontium Oxide Antibody-Modified IDE Surface

To check the limit of detection with SCC-Ag, different concentrations of SCC-Ag (100 fM to 100 nM) were added independently onto the strontium oxide antibody-modified IDE sensor surface. The current changes were noted before and after SCC-Ag immobilization. Between each immobilization, the surface was washed five times with PBS buffer to remove unbound SCC-Ag.

### 2.6. Specific Detection of SCC-Antigen on the Strontium-Antibody-Modified IDE Surface

To check the specific detection of SCC-Ag, after immobilizing the SCC-Ag antibody, the proteins (globulin; albumin) abundant in the blood serum and SCC-Ag were added independently, and the current changes were noted after the incubation, followed by washing as described above. Additionally, a control experiment was carried out with a different antibody (anti-factor IX) at 200 nM that was immobilized on the IDE substrate, followed by blocking with ethanolamine and the addition of 50 nM of SCC-antigen to check the specific detection of SCC-antigen.

## 3. Results and Discussion

**Figure 1:**
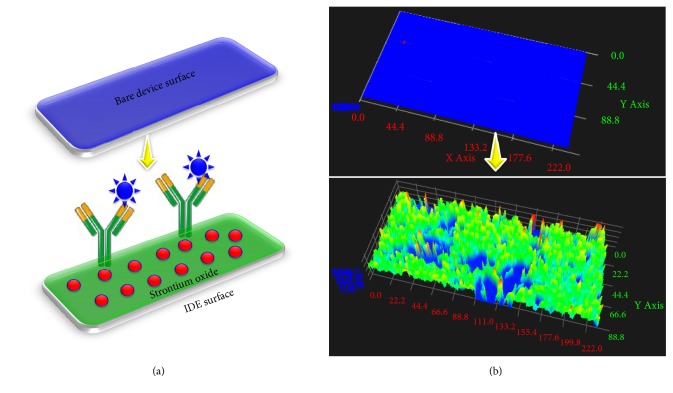
Surface of the interdigitated electrode. (a) Representation for the bare and modified sensing surface. (b) Three-dimensional images of the bare and modified sensing surface.

**Figure 2:**
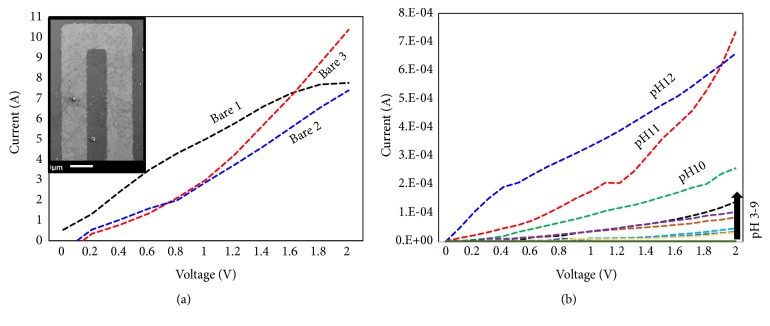
Surface characterization of IDE. (a) Reproducibility test on the bare devices by IDE. Three devices were tested. SEM image of the surface is displayed as the figure inset. (b) pH scouting. Different pH solutions from 1 to 12 were used.

Identifying and analyzing the condition and severity of diseases are mandatory in the field of medicine [30–32]. The biosensor is the established technology that helps to detect the basic to life-threatening diseases [33, 34]. Improving the biosensor obviously enhances the limit of detection with high-performance analysis. Immobilizing more biomolecules with the proper orientation on the sensing surface is one of the success keys to improving the limit of detection [35–38]. In this work, we used strontium oxide to immobilize the antibody on the IDE sensor surface to detect cervical cancer. This set up improved the antibody immobilization and limit of detection of SCC-Ag. The schematic representation of the attachment of strontium oxide and antibody for the interaction of SCC-Ag is displayed in Figure 1(a).  Figure 1(b) shows three-dimensional microscopic images of the bare surface between the fingered electrodes and strontium oxide-immobilized surface on the IDE sensor surface. The IDE surfaces were fabricated within the micron scale range with smooth and soft edges. The size of the gap between the fingers is ~5 *μ*m.  Figure 1(b) clearly shows the immobilization of strontium oxide on the IDE sensor surface, and there is an apparent difference from the bare device. On the strontium oxide-modified surface, the anti-SCC-Ag antibody was immobilized to detect SCC-Ag. The higher amount of antibodies was occupied on the IDE surface with the link of strontium oxide nanoparticles due to the enhanced surface area (Figure 1(b)), and it lowered the limit of detection of SCC-Ag. Before immobilizing the strontium on the IDE sensor surface, to check the reproducibility, three different batch preparations of IDE surfaces were checked with the IDE sensor.  Figure 2(a) shows the three different bare IDE sensor surfaces; all three batches showed a similar response of current changes without significant differences. Thus, the reproducibility of the IDE sensor surface was good. The surface morphology of the bare device was observed under scanning electron microscopy and is displayed in Figure 2(a) (inset).

### 3.1. pH Scouting on the IDE Surface

Because the solution conductivity is highly dependent on the dissociation of a dissolved substance in the solution in the form of ions, before detecting SCC-Ag, we checked the surface with different pH solutions to assess the capability of carrying the electric current. When the solution was added onto the electrode, the positive ions moved to the cathode and the negative ions moved toward the anode. We tried solutions with pH values from 1 to 12 on the IDE surface. In general, the acidic pH enhances the H+ ions and the alkaline pH yields more OH-ions in the solution. As shown in Figure 2(b), from pH 10, the current change was higher and the pH values from 3 to 9 show the lower current change. Apparently, pH values above 10 are considered to have fouling effect for biomolecular interaction. On the other hand, a pH until 9 falls under the condition with an insignificant noise or background level. From this result, we concluded that a pH range from 3 to 9 is suitable for our experiments, and we used the near neutral pH 7.4 for further experiments.

### 3.2. Detection of SCC-Ag on the Strontium Oxide Antibody-Modified Surface

**Figure 3:**
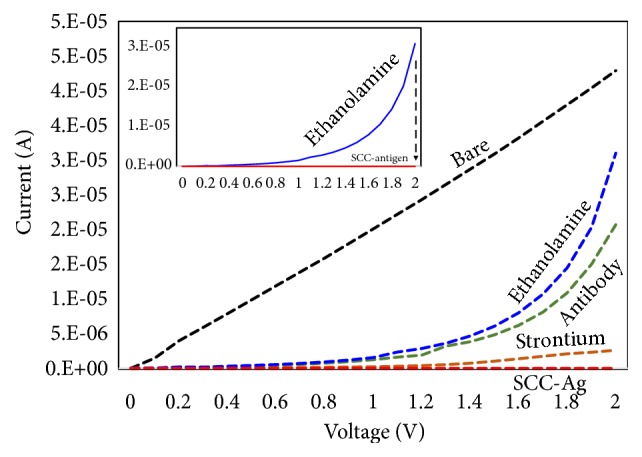
Molecular attachments on IDE. Different molecules were attached as per the scheme. It includes bare, strontium oxide, antibody, ethanolamine, and antigen.

After pH optimization, SCC-Ag was detected on the strontium oxide antibody-modified surface. To that end, 100 nM SCC-Ag was dropped onto the antibody immobilized surface.  Figure 3 shows the immobilization steps of each biomolecule on the IDE surface. The bare IDE (black line) shows the current approximately 4 ×10^−5^ in a linear fashion. After adding the APTES-modified strontium, the current was reduced to 3 ×10^−6^ (orange line). From these changes, we confirmed the immobilization of strontium oxide on the IDE surfaces and the declining change was due to the charged strontium oxide particle with APTES. Thereafter, when we added the antibody, it increased the current to 2 ×10^−5^, obviously in the reverse direction due to the opposite charge of the antibody compared with that of the strontium oxide with APTES. Next, we blocked the sensing surface with ethanolamine; the current was further increased to 3 ×10^−5^ in the same direction. These changes with the current confirmed the proper binding of the immobilized biomolecules on the sensing surface. Finally, when we added SCC-Ag, the current was decreased from 3 ×10^−5^ to the lowest current level. This is the indication of the binding of SCC-Ag with its antibody.

### 3.3. Limit of Detection of SCC-Ag

**Figure 4:**
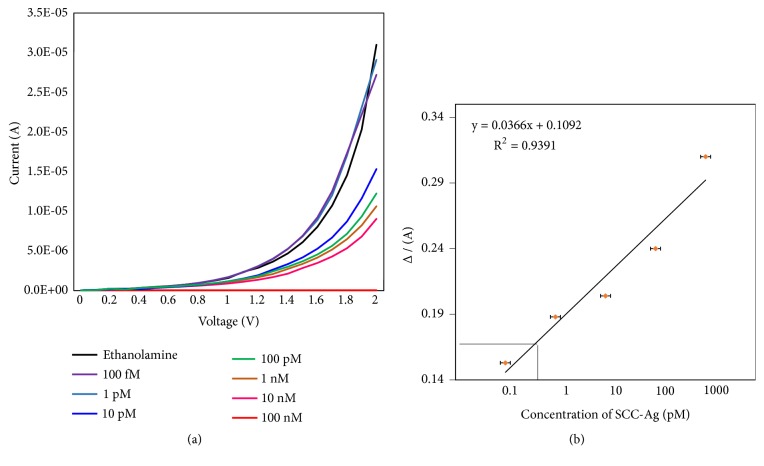
SSC-Ag analysis on the IDE. (a) Concentration-dependent analysis. The IDE surface was tested from 100 fM to 100 nM SSC-Ag. (b) Sensitivity analysis. The 3*σ* calculation was followed as indicated.

After detecting 100 nM of SCC-Ag on the strontium antibody-modified surface, to evaluate the limit of detection, we titrated SCC-Ag from the lower femtomolar (100 fM) until the nanomolar range (100 nM). After adding ethanolamine, 100 fM SCC-Ag was added, and the current change was noticed. Thereafter, upon adding each concentration from the above lowest to the highest (100 nM) concentration, the changes in the current were noted for each concentration. As shown in Figure 4(a), ethanolamine showed a current range >3 ×10^−5^. Upon adding different concentrations of SCC-Ag, notable changes were observed in the current. However, with SCC-Ag concentrations of 100 fM and 1 pM, no significant changes were found in the current and the responses were close to that of ethanolamine. Thereafter, when we added 10 pM SCC-Ag, the current level was decreased from 1.5 ×10^−5^. Additionally, when we increased the concentration, the changes in the current were also gradually decreased. From this result, we confirmed that the limit of detection of SCC-Ag on the strontium oxide antibody surface was 10 pM. The concentrations of 100 fM and 1 pM SCC-Ag did not display significant changes in the current. However, with 10 nM SCC-Ag, the current changed to 1.5 ×10^−5^, which could estimate the limit of detection. From the obtained data, the sensitivity of the device to detect SCC-Ag was calculated based on 3*σ* using ethanolamine followed by the buffer curve as the reference, and it was in the range between 1 and 10 pM.

### 3.4. Specific Detection of SCC-Ag

**Figure 5:**
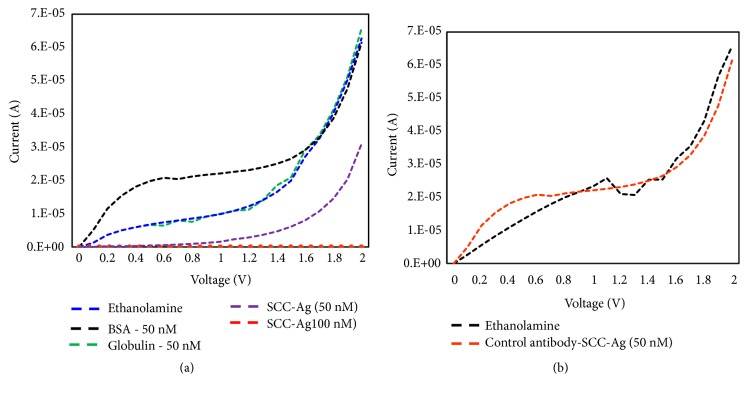
Specificity analysis on IDE. (a) Different serum proteins were tested at the 50-nM level. SSC-Ag, albumin, and globulin were tested. (b) Interaction of SCC-Ag against anti-factor IX antibody. The specificity of the anti-SCC antibody used in this study is clearly shown.

After checking the limit of detection, to evaluate the specific detection of SCC-Ag, upon immobilizing the ethanolamine, we dropped different unrelated proteins, including albumin and globulin, whose levels are abundant in human serum. Albumin in a normal human being is at the level of 45 mg mL^−1^, whereas that of globulin has been found to be 46 mg mL^−1^ [15]. As shown in Figure 5(a), after ethanolamine addition (the baseline), albumin and globulin showed no change in the current. Additionally, when we added SCC-Ag, a clear decrement was noted in the current with 50 nM SCC-Ag. This result indicated that SCC-Ag is specifically bound with its antibody and shows accurate detection of SCC-Ag. Moreover, to check the specificity, different antibodies (besides SCC antibody) were immobilized on the IDE substrate and were checked for interaction with SCC-antigen. As shown in Figure 5(b), SCC-antigen could not interact with factor IX antibody (no changes in the current), and this result confirmed that the SCC antibody and antigen interactions shown in study are specific. This research potentially addresses the current expectations in the field of medicine. Current identification methods are not sufficiently sensitive to quantify the lower level of SCC-Ag with higher nonfouling. With suitable surface functionalization using strontium oxide for probe immobilization, the detection level was improved and no significant biofouling was found [39, 40]. The primary advantage of our study understood completely the mechanism of surface chemical functionalization, especially using the strontium oxide-modified surface that is suitable for downstream applications. The stakeholders and potential users of the research proposed include researchers, the local population, and medical practitioners. Due to the early diagnosing strategy with a higher specificity, the system demonstrated here will aid in precautionary actions against further spreading squamous cell carcinoma in the human physiological system.

## 4. Conclusion

Cervical cancer is a life-threatening disease, and it affects the women's health. Squamous cell carcinoma antigen (SCC-Ag) is a well-known biomarker for cervical cancer. Early detection of cervical cancer using a suitable probe has improved the cure rate. In this work, we prepared a strontium oxide-modified IDE sensor surface to detect SCC-Ag against its antibody as the probe. The limit of detection was found at 10 pM, and the sensitivity range was from 1 to 10 pM. Moreover, a specific detection experiment was carried out using the serum proteins albumin and globulin, as well as different antibodies. SCC-Ag was demonstrated to be specifically detected on the strontium oxide-modified IDE sensor surface. This research demonstrates an efficient method to detect SCC-Ag that can be used for other disease biomarker detection.

## Data Availability

The data used to support the findings of this study are available from the corresponding author upon request.
